# Discovery of a Cushing’s syndrome protein kinase A mutant that biases signaling through type I AKAPs

**DOI:** 10.1126/sciadv.adl1258

**Published:** 2024-02-21

**Authors:** Mitchell H. Omar, Dominic P. Byrne, Safal Shrestha, Tyler M. Lakey, Kyung-Soon Lee, Sophia M. Lauer, Kerrie B. Collins, Leonard A. Daly, Claire E. Eyers, Geoffrey S. Baird, Shao-En Ong, Natarajan Kannan, Patrick A. Eyers, John D. Scott

**Affiliations:** ^1^ Department of Pharmacology, University of Washington, Seattle, WA 98195, USA.; ^2^ Department of Biochemistry, Cell and Systems Biology, University of Liverpool, Liverpool L69 7ZB, UK.; ^3^ Department of Biochemistry and Molecular Biology, University of Georgia, Athens, GA 30602, USA.; ^4^ Centre for Proteome Research, Department of Biochemistry, Cell and Systems Biology, University of Liverpool, Liverpool L69 7ZB, UK.; ^5^ Department of Laboratory Medicine and Pathology, University of Washington, Seattle, WA 98195, USA.

## Abstract

Adrenal Cushing’s syndrome is a disease of cortisol hypersecretion often caused by mutations in protein kinase A catalytic subunit (PKAc). Using a personalized medicine screening platform, we discovered a Cushing’s driver mutation, PKAc-W196G, in ~20% of patient samples analyzed. Proximity proteomics and photokinetic imaging reveal that PKAc^W196G^ is unexpectedly distinct from other described Cushing’s variants, exhibiting retained association with type I regulatory subunits (RI) and their corresponding A kinase anchoring proteins (AKAPs). Molecular dynamics simulations predict that substitution of tryptophan-196 with glycine creates a 653–cubic angstrom cleft between the catalytic core of PKAc^W196G^ and type II regulatory subunits (RII), but only a 395–cubic angstrom cleft with RI. Endocrine measurements show that overexpression of RIα or redistribution of PKAc^W196G^ via AKAP recruitment counteracts stress hormone overproduction. We conclude that a W196G mutation in the kinase catalytic core skews R subunit selectivity and biases AKAP association to drive Cushing’s syndrome.

## Teaser

The Cushing’s syndrome mutant PKAc-W196G drives disease despite retained association with type I regulatory complexes.

## INTRODUCTION

Hormones are chemical signals that travel through the bloodstream to control physiological processes in other parts of the body. Within target cells, hormone action often proceeds through generation of tightly regulated second messengers such as inositol phosphate or cyclic adenosine 3′,5′-monophosphate (cAMP) (*1*, *2*). Elegant live-cell studies and the discovery of intracellular G protein–coupled receptor signaling have redefined our understanding of cAMP action, which we now know is constrained to nanometer-scale signaling compartments (*3*–*7*). This cAMP nanodomain model is congruent with molecular evidence that its major downstream effector protein kinase A (PKA) operates within a similar radius of action (*8*, *9*).

PKA is a highly used kinase capable of phosphorylating hundreds of protein substrates (*10*). Such functional diversity is achieved via two principal factors: (i) differing sensitivity to cAMP, as determined by type I or type II regulatory subunits (RI and RII, respectively); and (ii) subcellular targeting via distinct A kinase anchoring proteins (AKAPs) (*11*). AKAPs are especially crucial as they maintain the integrity of PKA signaling islands and delineate the numerous roles of this promiscuous kinase (*11*–*14*). Local activation of anchored PKA explains how individual hormones can simultaneously propagate discrete cAMP responses in the same cell (*5*, *11*).

In the human stress response, changes to organ systems are governed by the secretion of catecholamine and glucocorticoid hormones from the adrenal glands (*15*). Cortisol release from zona fasciculata cells in the adrenal cortex triggers many vital physiological processes, such as increasing blood glucose availability, enhancing catabolism of protein and fat, and down-regulating inflammatory pathways (*2*, *15*, *16*). However, sustained exposure to cortisol has adverse effects. These include weight gain, cardiovascular disease, elevated blood glucose, and neuropsychiatric manifestations (*17*). In the chronic stress disorder Cushing’s syndrome, cellular processes are dysregulated by increased exposure to stress hormones. This can result from exogenous causes, such as high levels of anti-inflammatory medications, or endogenous overproduction of cortisol (*18*). Among the endogenous cases, adrenocorticotropic hormone (ACTH)–dependent Cushing’s syndrome (also known as Cushing’s disease) emanates from pituitary tumors. ACTH-independent cases (also called adrenal Cushing’s syndrome) can develop from adenomas, hyperplasia, or micronodular dysplasia of the adrenal glands (*17*).

Somatic mutations in PKA catalytic subunit (PKAc) underlie adrenal Cushing’s syndrome (*16*, *19*–*23*). The most prevalent mutant, PKAc-L205R, is found in ~45% of patients with Cushing’s syndrome, while other PKAc mutations have been reported at frequencies of less than 1% (*24*). These disease-causing kinase variants diverge in protein stability, levels of autophosphorylation, subcellular distribution, and their engagement with downstream signaling pathways (*25*, *26*). Cushing’s PKAc variants can also be classified on the basis of differential association with the endogenous heat-stable inhibitor PKI (*26*). This suggests that the mechanisms of action of individual Cushing’s kinases may be distinctive. We previously reported that the added charge of Arg^196^ perturbs the topology of PKAc^W196R^ in a manner that mobilizes ERK signaling cascades. Unlike Arg^205^, this mutation does not compromise catalytic efficiency of the kinase or disrupt interactions with PKI (*25*, *26*). Here, we report the discovery of a genetic Cushing’s variant that introduces glycine at position 196 in the catalytic core of the kinase. PKAc^W196G^ is distinct from other Cushing’s mutants, including PKAc^W196R^, due to a different subcellular distribution in adenomas and a preferential association with RI. Molecular modeling predicts that replacing tryptophan-196 with glycine creates packing defects in the kinase that disrupt catalytic subunit association with RII but preserve its affinity toward RI. Functional studies confirm this prediction showing that overexpression of RIα or recruitment of PKAc^W196G^ into type I AKAP signaling islands corrects stress hormone hypersecretion. Thus, structural perturbation in the activation loop of PKA influences R subunit selectivity and location rather than catalytic efficacy to enhance the pathological impact of this Cushing’s variant.

## RESULTS

### PKAc-W196G underlies adrenal Cushing’s syndrome

**Fig. 1.**
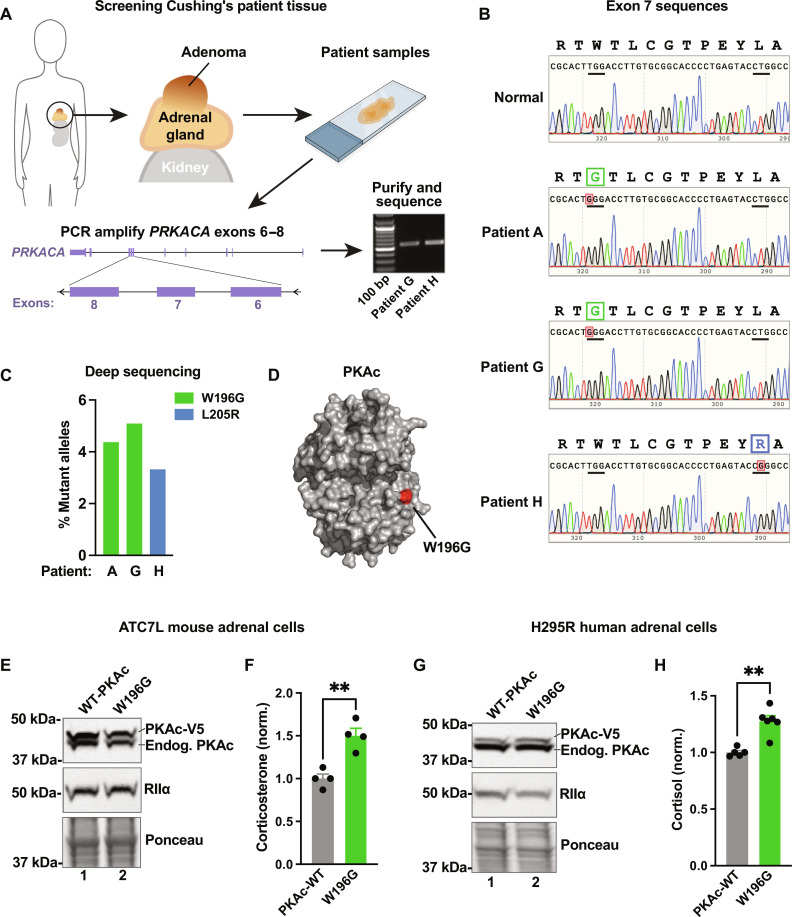
Discovery of adrenal Cushing’s syndrome mutation PKAc-W196G. (**A**) Workflow for screening Cushing’s syndrome patient samples. (**B**) *PRKACA* sequencing traces (amino acids194 to 206) for normal tissue (top) and three patient tumors with PKAc missense mutations. (**C**) Quantitation of deep sequencing reads for the three patient tumors. Data represented as percentage from up to 300,000 reads. (**D**) Protein structure model detailing PKAc-W196G mutation. Glycine-196 (red) is indicated. Derived from Protein Data Bank file 6E99. (**E**) Immunoblot of ATC7L lysates after infection with RIIα and either wild-type (WT) PKAc (lane 1) or PKAc-W196G (lane 2). (**F**) ATC7L cell corticosterone measurements. Means ± SE; *n* = 4; **P ≤ 0.01, Student’s *t* test. (**G**) Immunoblot of H295R lysates after infection with RIIα and either WT PKAc (lane 1) or PKAc-W196G (lane 2). (**H**) H295R cell cortisol measurements. Means ± SE; *n* ≥ 5; **P ≤ 0.01, Student’s *t* test.

Single-allele mutations in the *PRKACA* gene drive adrenal Cushing’s syndrome (*25*). Given the incidence of this endocrine disorder, we reasoned that additional Cushing’s mutations must exist. Therefore, we designed a personalized medicine workflow to match mutant PKAc alleles with anonymized age- and gender-matched adrenal Cushing’s patient samples. Most described Cushing’s mutations occur within exon 7 of *PRKACA* (*24*, *27*). DNA was extracted directly from fixed pathological sections, and exons 6 to 8 of the *PRKACA* gene were amplified by polymerase chain reaction (PCR) (Fig. 1A). Upon sequencing purified DNA with standard Sanger methods, the L205R (c.617A>C) mutation was identified in patient H (Fig. 1B, bottom panel). Unexpectedly, adenomas from patients A and G harbored a previously unidentified Cushing’s PKAc variant with a W196G (c.589A>C) point mutation (Fig. 1B, middle panels). We further validated the presence of these alleles in patient tumors using next-generation deep sequencing (Fig. 1C). Reads at a depth of >150,000 per sample confirmed that PKAc^W196G^ was the predominant mutant allele in patient A (4.4%) and patient G (5.1%; Fig. 1C). The tumor from patient H harbored a PKAc^L205R^ allele (3.3%; Fig. 1C). Molecular modeling demonstrates that the W196G substitution occurs within the activation segment on the catalytic subunit, adjacent to the binding site for substrates and R subunits (Fig. 1D). The sufficiency of PKAc^W196G^ to drive stress hormone production was investigated in two adrenal cell lines (Fig. 1, E to H). We virally expressed mutant kinase in mouse ATC7L (Fig. 1E) and human NCI-H295R (Fig. 1G) cell lines and measured stress hormone release by enzyme-linked immunosorbent assay (ELISA) (Fig. 1, F and H). In both cases, glucocorticoid production in the presence of PKAc^W196G^ was elevated over wild-type (WT) PKAc controls. Together, our personalized medicine screening and endocrine profiling indicates that the PKAc^W196G^ mutant drives adrenal Cushing’s syndrome pathology.

### Patient tissue analyses reveal a distinct subcellular distribution of PKAc^W196G^

**Fig. 2.**
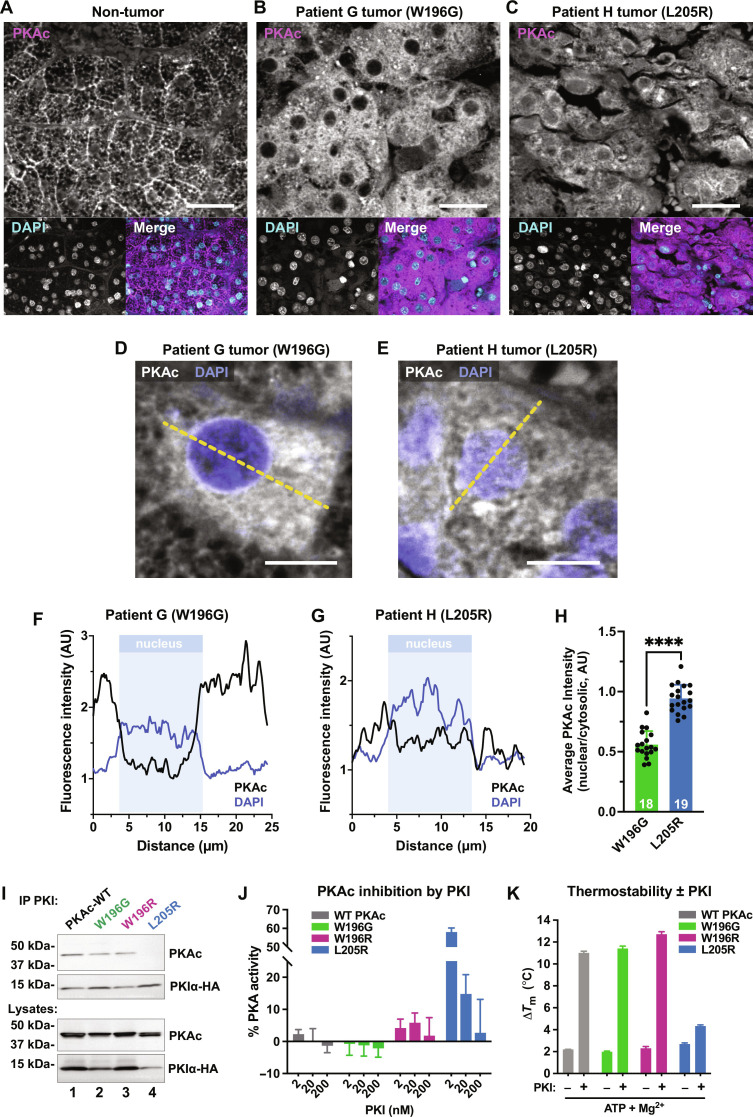
Patient tissue analyses reveal a distinct subcellular distribution of PKAc^W196G^. (**A** to **C**) Patient adrenal tissue and Cushing’s adenomas stained for PKAc (magenta) and nuclei [4′,6-diamidino-2-phenylindole (DAPI), cyan]; patient G adjacent non-tumor (A), patient G tumor (B), and patient H tumor (C). Scale bars, 30 μm. (**D** and **E**) High magnification of patient tumors stained for PKAc (white) and nuclei (DAPI, blue); patient G (D), and patient H (E). Yellow dashed line represents measurements depicted in (F) and (G). Scale bars, 10 μm. (**F** and **G**) Line plot quantification of PKAc subcellular distribution. Example trace of PKAc (black) and DAPI (blue) signals versus distance. Nuclear bounds are indicated (pale blue shading); patient G, PKAc^W196G^ (F); patient H, PKAc^L205R^ (G). (**H**) Quantitation of PKAc intensity measurements, nuclear/cytosolic. Means ± SD; *n* ≥ 18; ****P ≤ 0.0001, Student’s *t* test. AU, arbitrary units. (**I**) Immunoprecipitation of PKI from human embryonic kidney (HEK) 293T cells expressing PKIα-HA along with V5-tagged PKAc variants: WT PKAc (lane 1), PKAc-W196G (lane 2), PKAc-W196R (lane 3), and PKAc-L205R (lane 4). Representative of three experimental replicates. (**J**) Inhibition of PKAc activity toward Kemptide peptide substrate relative to buffer controls for each variant upon increasing concentrations of PKI. Means ± SE; *n* = 3. (**K**) Thermostability measurements for each PKAc variant ± PKI. Experiments conducted in the presence and absence of Mg^2+^ adenosine 5′-triphosphate (ATP). Means ± SE; *n* = 3.

An advantage of obtaining 10 serial sections per patient was that it allowed further analyses of each Cushing’s sample. High-resolution immunofluorescence imaging of normal adrenal tissue revealed an accumulation of native PKAc at cell-cell junctions and an intracellular honeycomb pattern (Fig. 2A and fig. S1A). This organization is lost in tumors harboring W196G or L205R mutations (Fig. 2, B and C). In samples from patient G, PKAc^W196G^ exhibits a diffuse cytoplasmic distribution and exclusion from the nucleus (Fig. 2, B and D). In patient H, PKAc^L205R^ is uniformly dispersed across the cytoplasm and nucleus (Fig. 2, C and E). Quantitative line plot analyses confirmed that the PKAc-W196G and PKAc-L205R mutants exhibit distinct subcellular distributions in Cushing’s adenomas (Fig. 2, D to G). Measurements from multiple sections are quantified in Fig. 2H.

The subcellular distribution of PKAc^L205R^ depicted in Fig. 2 (C and E) is consistent with the inability of this Cushing’s variant to interact with R subunits (*25*, *26*). PKAc^L205R^ is also unable to interact with the endogenous heat-stable PKA inhibitor PKI (*26*, *28*). We have recently shown that certain Cushing’s PKAc variants interact with PKI and are shuttled back to the cytosol by a nuclear export sequence on the inhibitor (*26*, *29*, *30*). Immunoprecipitations from adrenal cells infected with Cushing’s variants demonstrated association of PKIα with WT PKAc, PKAc^W196G^, and PKAc^W196R^ (Fig. 2I, lanes 1 to 3). As expected, PKAc^L205R^ failed to coprecipitate with PKI (Fig. 2I, lane 4). Kinase activity measurements confirmed that the L205R catalytic domain was refractory to inhibition by PKI, whereas PKAc^W196G^ and PKAc^W196R^ were potently inhibited (Fig. 2J and fig. S1B). Thermal profiling of Cushing’s variants demonstrated that WT PKAc, PKAc^W196G^, and PKAc^W196R^ have similar high melting temperatures upon coincubation with Mg–adenosine 5′-triphosphate (ATP) and PKI, with melting temperature (*T*_m_) values elevated by 8° to 10°C (Fig. 2K). In contrast, the thermostability of PKAc^L205R^ was minimally affected by PKI (Fig. 2K, blue). These studies indicate that PKAc^W196G^ is physiochemically more similar to the native enzyme than to PKAc^L205R^.

### PKAc^W196G^ is differentially compartmentalized in adrenal cells

**Fig. 3.**
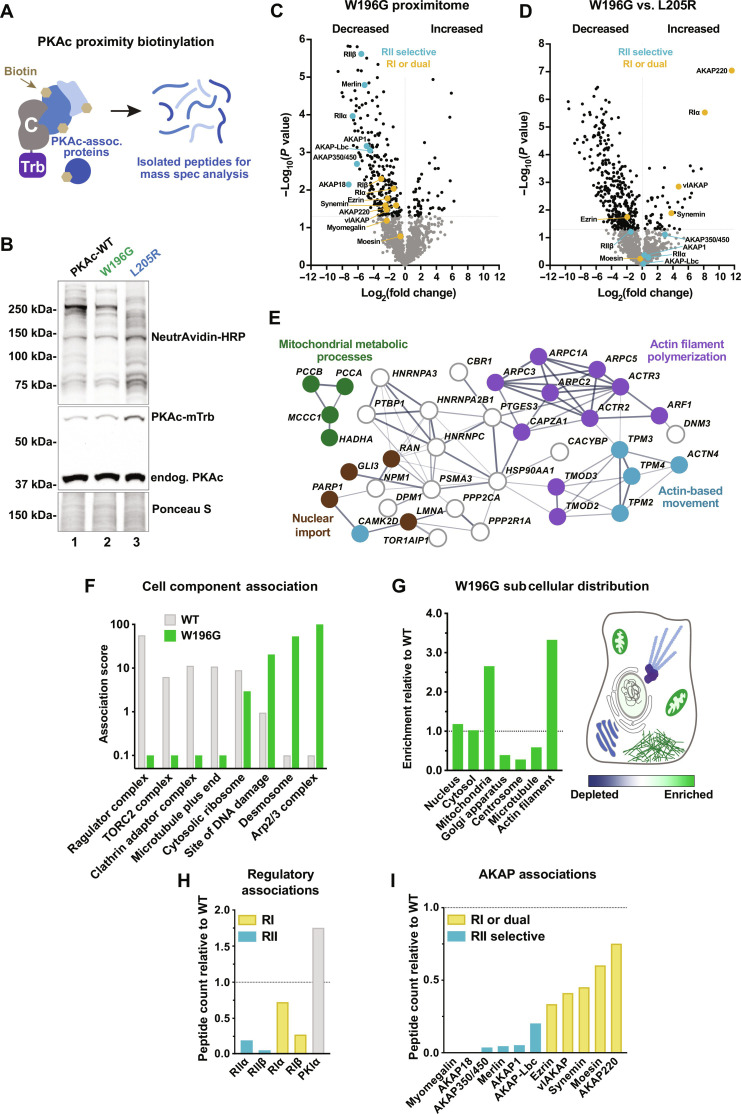
PKAc^W196G^ is differentially compartmentalized in adrenal cells. (**A**) Diagram depicts biotin-labeled proteins surrounding PKAc-miniTurbo in live cells and the final isolated peptides after streptavidin capture and trypsin digest. Labels: C, PKAc; Trb, miniTurbo. (**B**) Immunoblot of lysates from stable PKAc-miniTurbo H295R cell lines upon 48 hours of doxycycline induction and 2 hours of biotin incubation. NeutrAvidin-HRP labels biotinylated proteins. Expression of each PKAc-miniTurbo variant is indicated above each lane. Representative of four biological replicates. (**C** and **D**) Volcano plots of proximity proteomics for PKAc-W196G versus WT PKAc (C) and PKAc-W196G versus PKAc-L205R (D). Proteins underrepresented (left) and enriched (right) in the PKAc^W196G^ proximity proteome. Gray dots indicate proteins with a corrected *P* value lower than 0.05. Colored dots indicate RII-selective (teal) and RI-recruiting (yellow) complex components. Data from four biological replicates. (**E**) STRING network depiction of proteins with increased PKAc^W196G^ association versus WT PKAc. (**F**) Selected gene ontology cell component enrichment scores for highly disparate categories between WT PKAc (gray) and PKAc-W196G (green). (**G**) Left: PKAc-W196G gene ontology enrichment for major cell compartments and organelles relative to WT PKAc. Right: Schematic depiction of results in a prototypic cell. (**H**) PKAc-W196G association with PKA regulatory components relative to WT PKAc. (**I**) PKAc-W196G association with AKAPs relative to WT PKAc. Colors indicate likely RII-selective (teal) and RI-recruiting (yellow) AKAPs.

We next took an unbiased approach to identify interacting proteins and signaling partners for PKAc^W196G^ (*31*). Proximity biotinylation was performed in stable H295R adrenal cell lines that inducibly expressed the promiscuous biotin ligase miniTurbo fused to the c-terminus of PKAc (Fig. 3A). Levels of tagged WT PKAc, PKAc^W196G^, and PKAc^L205R^ were optimized for subtle expression compared to that of endogenous PKAc (Fig. 3B, middle panel). Biotinylation was verified by NeutrAvidin–horseradish peroxidase (HRP) staining (Fig. 3B, top panel). Liquid chromatography–tandem mass spectrometry (LC-MS/MS) identified 1359 proteins proximal to PKAc among all samples. Quantitative analysis revealed that the proximity of 34 proteins significantly increased for the W196G variant compared to that for WT PKAc, while 285 proteins decreased for the mutant kinase (Fig. 3C). Comparison of PKAc^W196G^ with PKAc^L205R^ identified 45 proteins more strongly associated with W196G, compared to 306 proteins less associated with this Cushing’s variant (Fig. 3D). Search tool for recurring instances of neighbouring genes (STRING) network analysis of proteins with increased proximity to PKAc-W196G versus WT PKAc highlighted functional clusters involved in actin cell biology, nuclear import, and mitochondrial metabolic processes (Fig. 3E) (*32*). Protein analysis through evolutionary relationships (PANTHER)-based gene ontology cell component analyses identified an increase of PKAc^W196G^ association with sites of DNA damage, desmosomes, and the Arp2/3 complex (Fig. 3F, green) (*33*). Further analyses indicated that proteins proximal to PKAc^W196G^ are enriched at mitochondria and actin filaments but are depleted at the Golgi apparatus, centrosome, and microtubules (Fig. 3G).

Mining our proteomic datasets for PKA subunits and AKAPs revealed an unexpected finding. While type II PKA regulatory subunits and RII-selective AKAPs were depleted from the PKAc^W196G^ proximity proteome (Fig. 3, H and I, teal), this Cushing’s mutant maintained associations with RI and their AKAPs (Fig. 3, H and I, yellow). In addition, the PKAc^W196G^ proteomic dataset was notably more depleted in anchored type II PKA components (teal dots) than type I components (yellow dots) when normalized to the WT kinase (Fig. 3C). In addition, when comparing PKAc^W196G^ with PKAc^L205R^, type I PKA signaling components were among the most significantly enriched hits for PKAc^W196G^ (Fig. 3D, yellow dots). Together, these data suggest that the W196G Cushing’s syndrome mutation selectively preserves RI association with the catalytic subunit.

### Molecular dynamics offer a mechanistic explanation for RI selectivity of PKAc^W196G^

**Fig. 4.**
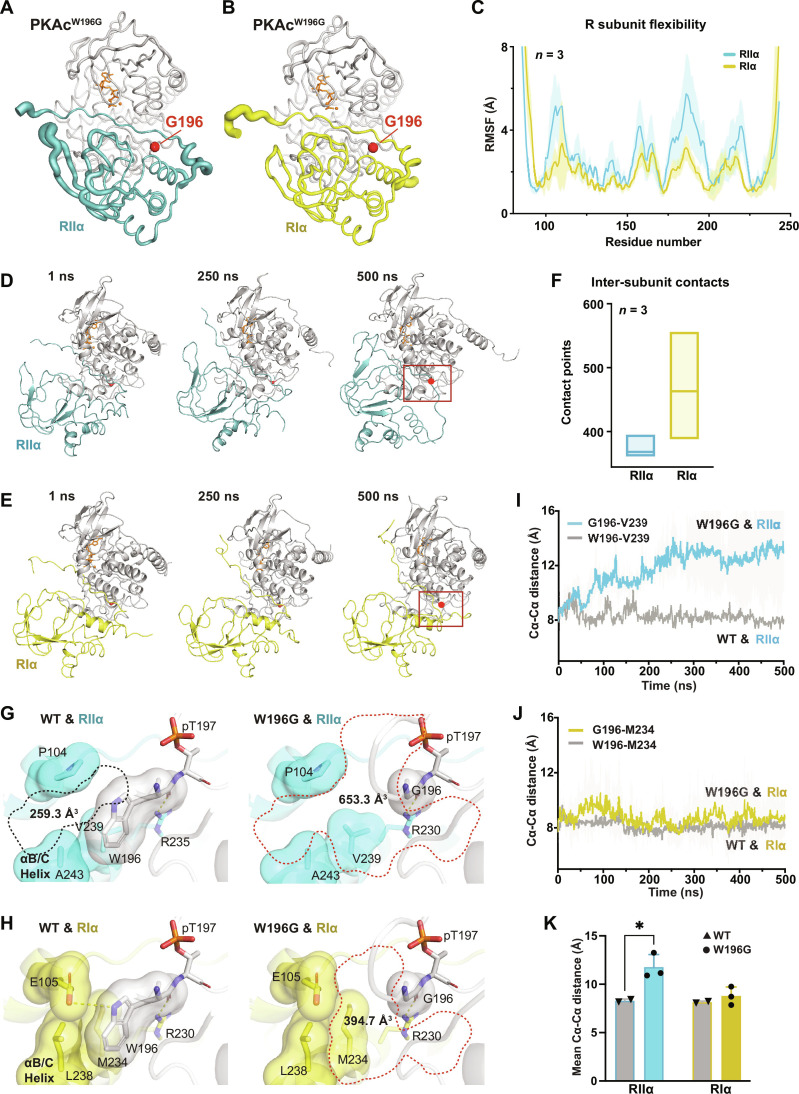
A mechanistic explanation for R selectivity of PKAc^W196G^. (**A** and **B**) B-factor putty diagram of PKAc^W196G^ complexed with RIIα (A) or RIα (B). Thicker lines represent higher fluctuations. ATP and magnesium ions are shown in orange. (**C**) Plot showing the root mean square fluctuation (RMSF) values of RIIα (teal) and RIα (yellow) subunits plotted against residue number when complexed with PKAc^W196G^. Fluctuations were calculated for Cα atoms over the entire trajectory. Means ± SD; *n* = 3 independent simulations. (**D** and **E**) Molecular dynamics (MD) time-course montages for PKAc^W196G^ complexed with RIIα (D) and RIα (E). Red dot indicates glycine-196. Box indicates regions expanded and featured in (G) and (H). (**F**) Box plot showing the total number of contacts made during the 500-ns simulation between PKAc-W196G and each regulatory subunit. PyContact was used to calculate the total number of contacts. *n* = 3 independent simulations. (**G** and **H**) Cavities (dotted lines) at the interface between PKA catalytic and either RIIα (G) or RIα (H) regulatory subunits. Key residues at interfaces are shown as both sticks and surface representations. Polar contacts are indicated by yellow dashed lines. Cavity volumes were calculated using F pocket version 3.0. Representative of three independent replicates. (**I**) Plot of the Cα-Cα distance between V239 in RIIα and residue 196 in PKAc variants. PKAc^W196G^ (teal; *n* = 3) experiences much greater displacement from RIIα than does the WT kinase (gray; *n* = 2) over 500-ns simulations. Means ± SD. (**J**) Plot of the Cα-Cα distance between M234 in RIα and residue 196 in PKAc variants. PKAc^W196G^ (yellow; *n* = 3) experiences the same displacement as the WT kinase (gray; *n* = 2) over 500-ns simulations. (**K**) Bar graph of the 500-ns time points from (I) and (J). Means ± SE. *P ≤ 0.05, Student’s *t* test.

Our proximity proteomics data suggest that type I PKA signaling predominates in Cushing’s adenomas expressing PKAc^W196G^. To investigate the mechanisms underlying this selectivity, we performed molecular dynamics (MD) simulations of PKAc^W196G^ complexed with RI or RII (Fig. 4, A and B) (*34*). Analysis of thermal fluctuations revealed increased flexibility of RIIα (teal) compared to RIα (yellow). This is depicted by thicker lines in PyMOL putty representations (Fig. 4, A and B). Quantitative differences between the R subunits are shown as root mean square fluctuations plotted against residue number (Fig. 4C). MD time courses and quantitative analysis of contact points between PKAc^W196G^ and regulatory subunits from three simulations reveal a less stable protein complex when this Cushing’s variant is paired with RIIα (Fig. 4, D to F, and fig. S2, A and B).

RII thermal fluctuations increased with time, resulting in changes in hydrogen bonding and packing interactions at the RIIα:PKAc^W196G^ interface (fig. S2C). Structural perturbations were much less evident in the RIα:PKAc^W196G^ interface (fig. S2D). At the atomic level, tryptophan-196 of WT PKAc mediates key packing interactions with hydrophobic and polar residues on the regulatory subunit (*35*). In the W196G Cushing’s mutant, glycine-196 creates a cleft in the R subunit interface, as indicated by increased cavity volumes relative to the WT kinase (Fig. 4, G and H). These packing defects are significantly larger in the RIIα:PKAc^W196G^ interface as compared to that in RIα. Such topological differences may be a consequence of sequence variation between the two isoforms. Specifically, Val^239^, Ala^243^, and Pro^105^ in RIIα create a cavity of 653.3 Å^3^ at the interface with PKAc^W196G^ (Fig. 4G, right panel). The corresponding cleft between WT PKAc and RIIα is only 259.3 Å^3^ (Fig. 4G, left panel) (*36*). In RIα, there is tight packing of Met^234^, Leu^238^, and Glu^105^ with WT PKAc (Fig. 4H, left panel), and introduction of glycine-196 only creates a 394.7-Å^3^ gap at the RIα:PKAc^W196G^ interface (Fig. 4H, right panel).

This suggested the possibility of larger destabilization of RIIα interactions relative to RIα interactions by the Cushing’s variant. Analysis of backbone flexibility at the R:C interface using Cα-Cα distances between either tryptophan- or glycine-196 of PKAc and proximal residues in the regulatory subunits (Val 239^RIIα^ and Met 234^RIα^) indicates a progressive increase in Cα-Cα distances in the RIIα:PKAc^W196G^ simulations relative to WT complexes (Fig. 4I). In contrast, the Cα-Cα distances are comparable for WT PKAc and the W196G mutant in RIα:PKAc simulations (Fig. 4J). Together, these data suggest that introduction of glycine-196 in the catalytic subunit selectively destabilizes interactions with RIIα while minimally affecting autoregulation and anchoring by RIα (Fig. 4, I to K).

### PKAc^W196G^ activity is preferentially inhibited by RI

**Fig. 5.**
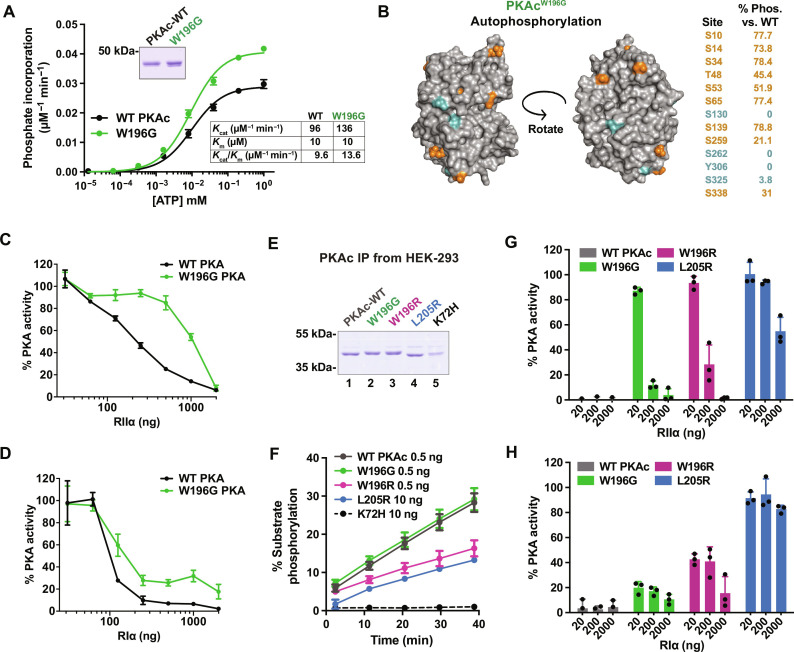
PKAc^W196G^ is preferentially inhibited by RI subunits. (**A**) Top inset: Coomassie blue–stained SDS–polyacrylamide gel electrophoresis (PAGE) of bacterially purified recombinant WT PKAc and PKAc^W196G^. Graph: PKA-catalyzed Kemptide phosphorylation (pmol phosphate/min per ng protein) with increasing concentrations of ATP, normalized to the maximum rate of phosphorylation for each protein. Lower inset: *K*_cat_, *K*_m[ATP]_, and *K*_cat_/*K*_m[ATP]_ determination for WT and mutant PKAc. Means ± SD; *n* = 4 independent experiments. (**B**) Structural depictions and quantification of phosphorylated sites on bacterially purified PKAc^W196G^ as measured by quantitative MS. Orange > 21%, turquoise < 4% phosphorylation as compared to WT PKAc levels. Average data from three independent replicates. (**C** and **D**) PKAc kinase activity measurements in the presence of RIIα (C) or RIα (D) shown as % buffer control for WT PKAc (black) and PKAc^W196G^ (green). Kinase activity measurements used Kemptide as a substrate. Means ± SD; *n* = 4. (**E**) Coomassie blue staining of SDS-PAGE showing PKAc and Cushing’s variants purified from HEK-293T cells by immunoprecipitation. (**F**) Real-time measurements of Kemptide phosphorylation among HEK-293T–purified PKAc variants. L205R and K72H mutants were used at 20× concentration for presentation purposes. Means ± SD; *n* = 3. (**G** and **H**) PKAc activity toward Kemptide relative to buffer controls for each HEK-293T–purified variant upon increasing concentrations of RIIα (G) or RIα (H). Means ± SD; *n* = 3.

Our structural evaluation predicted physiochemical differences between WT PKAc and the W196G Cushing’s mutant. Catalytic subunits were expressed and purified from bacteria, and kinase activity was measured at increasing concentrations of ATP using Kemptide as a substrate (Fig. 5A). Both PKAc forms exhibited similar *K*_cat_ (PKAc, 96 ± 1.5 μM/min; W196G, 136 ± 1.8 μM/min) and *K*_m[ATP]_ values (PKAc, 10 ± 0.8 μM; W196G, 10 ± 0.7 μM). Autophosphorylation of the purified catalytic subunits was measured by quantitative MS after proteolysis. Thirteen autophosphorylation sites were decreased on PKAc^W196G^ compared to WT kinase (Fig. 5B). Of these, nine sites exhibited between 20 and 80% occupancy of phosphorylation levels quantified for the WT kinase (Fig. 5B, orange). Phosphate incorporation was negligible for the other four sites (Fig. 5B, turquoise). On the basis of our modeling data, we reasoned that such changes in the catalytic core of the W196G mutant would affect regulation of this Cushing’s mutant by R subunits. Enzyme assays demonstrated reduced inhibition of the W196G variant by titrated RIIα as compared to WT PKAc (Fig. 5C). In contrast, RIα readily inhibited PKAc^W196G^ (Fig. 5D).

Mammalian and bacterial protein synthesis pathways differ in protein folding, processing, and post-translational modification (*37*). Therefore, we next profiled the activities of Cushing’s PKAc variants purified from human embryonic kidney (HEK) 293T cells (Fig. 5, E and F). PKAc^W196G^ and WT PKAc exhibited identical Kemptide phosphorylation rates (Fig. 5F, green versus charcoal). In contrast, the W196R analog and PKAc^L205R^ are less catalytically efficient toward this substrate (Fig. 5F, magenta and blue), while a kinase-dead PKAc^K72H^ control had no catalytic activity (Fig. 5F, black). PKAc activity measurements also confirmed decreased RIIα autoinhibition of all Cushing’s catalytic domain variants, as compared to the WT kinase (Fig. 5G). Parallel experiments with RIα measured strong inhibition of PKAc^W196G^, moderate inhibition of PKAc^W196R^, and no inhibition of the L205R mutant (Fig. 5H). MS analyses of PKAc^W196G^ and PKAc^W196R^ complexes immunoprecipitated from HEK-293T cells also corroborated a strong preference for RIα association compared to interaction with RIIα (fig. S3). Control experiments confirmed that PKAc^L205R^ exhibited negligible associations with either regulatory subunit (fig. S3).

### PKAc^W196G^ is recruited into type I AKAP complexes

**Fig. 6.**
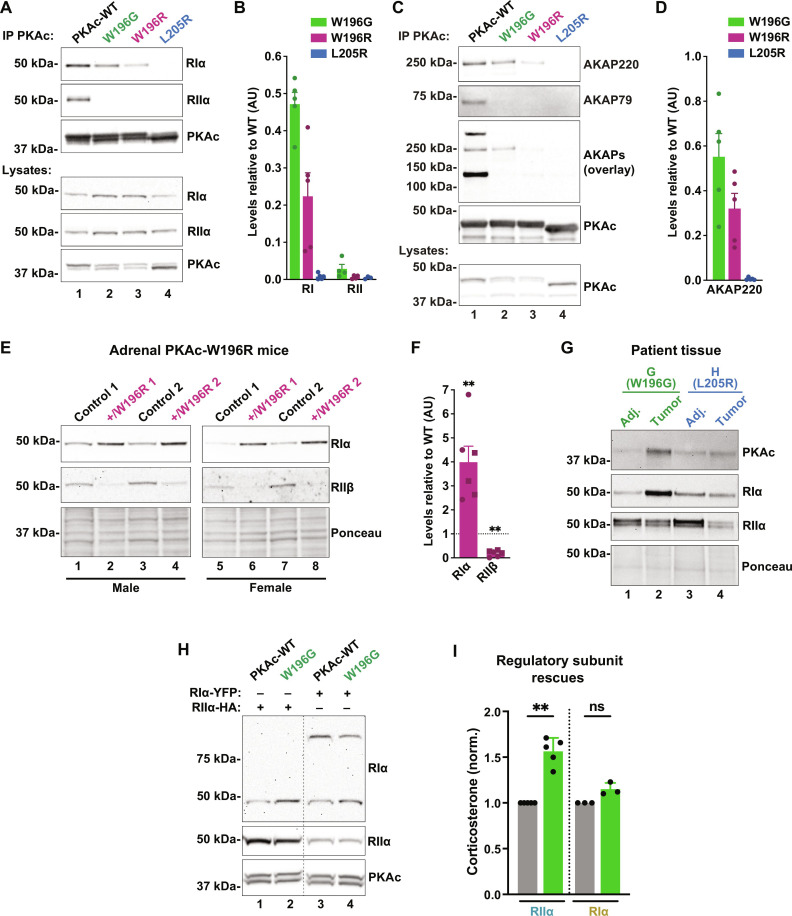
PKAc^W196G^ is recruited into type I AKAP complexes. (**A**) Immunoprecipitation of V5-tagged PKAc variants from H295R adrenal cells demonstrating coprecipitation of RIα (top panel) and RIIα (second panel). Representative of four replicates. (**B**) Quantitation of immunoblot intensity for RIα (left) and RIIα (right) coimmunoprecipitation relative to WT PKAc. Means ± SE; *n* ≥ 4. (**C**) Immunoprecipitation of V5-tagged PKAc variants from H295R adrenal cells demonstrating coprecipitation of the dual-specific AKAP220 (top panel) and the RII-selective AKAP79 (second panel). Representative of four replicates. (**D**) Quantitation of AKAP220 coprecipitation by Cushing’s mutants relative to WT PKAc. Means ± SE; *n* = 5. (**E**) Immunoblot of RIα (top panel) and RIIβ (second panel) levels in adrenal lysates from adrenal-specific PRKACA^+/W196R^ heterozygous mice, an animal model of adrenal Cushing’s syndrome (*25*). Sex-matched littermates were used as controls for male (lanes 1 to 4) and female (lanes 5 to 8) mutant mice. Ponceau S staining (bottom panel) served as a loading control. (**F**) Quantification of RIα and RIIβ protein levels from (E). Means ± SE; *n* = 6; **P ≤ 0.01, Student’s *t* test. (**G**) Immunoblot analysis of PKA Cα (top), RIα (top middle), and RIIα (bottom middle) subunit levels in adrenal tissue lysates from patients with Cushing’s syndrome. Adj, tumor-adjacent tissue. Ponceau S staining (bottom panel) served as a loading control. (**H**) Immunoblot of ATC7L cells virally infected with either WT or W196G PKAc and either RIα–yellow fluorescent protein (YFP) or RIIα–hemagglutinin (HA). Doublet for PKAc shows V5-tagged (top) and endogenous (bottom) kinase. Representative of at least three biological replicates. (**I**) Corticosterone measurements from conditions shown in (H). Excess RIα rescues hormone overproduction by PKAc^W196G^ expressing cells. Means ± SD; *n* ≥ 3; **P ≤ 0.01, analysis of variance (ANOVA) with Dunn’s multiple comparisons.

Immunoprecipitation of Cushing’s variants from adrenal cells also revealed differential association with RI (Fig. 6A). WT PKAc association with RI and RII served as a positive control (Fig. 6A, lane 1). PKAc^W196G^ bound RIα more avidly than the W196R or L205R mutants, and none of the Cushing’s variants coprecipitated RII under the same conditions (Fig. 6, A, top panels, and B). RI is recruited to type I and dual-selectivity anchoring proteins such as AKAP220 (*38*–*41*). This anchoring protein was readily detected upon precipitation of PKAc^W196G^, weakly detected with the W196R analog, and absent in PKAc^L205R^ immune complexes (Fig. 6, C, top panel, and D). In contrast, the type II–selective anchoring protein AKAP79 did not coprecipitate with any Cushing’s variant (Fig. 6C, second panel). Broad detection of type II AKAPs was achieved using an RII overlay (Fig. 6C, middle panel) (*42*).

In comparison to type II PKA, the type I holoenzyme is activated at sevenfold lower levels of second messenger (*43*–*45*). Thus, an increased RI to RII ratio should sensitize tumors to cAMP. We detected enhanced stabilization of RIα in mice heterozygous for the PKA^W196R^ Cushing’s mutation in the adrenal cortex (*25*). Immunoblot analyses of adrenal gland lysates from both male and female mutant mice demonstrated a fourfold up-regulation of RIα levels and a concomitant decrease in RII levels (Fig. 6, E and F). This finding translated to Cushing’s syndrome patient samples (Fig. 6G). Adenomas from patients with the W196G mutation demonstrated elevated RIα levels, while tumor samples from the patient harboring an L205R mutation did not (Fig. 6G). These clinical results are consistent with a preferential stabilization of RI subunits by PKAc^w196G^. We next tested the hypothesis that increased RI levels moderate the pathological impact of PKAc^W196G^ in Cushing’s syndrome. ATC7L cells were infected with either WT PKAc or the W196G variant. Both cell lines were also infected with vectors expressing RI–yellow fluorescent protein (YFP) or hemagglutinin (HA)–tagged RII, resulting in a twofold excess of the respective regulatory subunit (Fig. 6H). Corticosterone release was measured by ELISA (Fig. 6I). While overexpression of RIIα could not suppress corticosterone synthesis, excess RIα restored stress hormone production to WT levels (Fig. 6I). Thus, autoregulation by RI attenuates the pathological influence of the W196G Cushing’s kinase.

### Sequestrating PKAc^W196G^ corrects cortisol overproduction

**Fig. 7.**
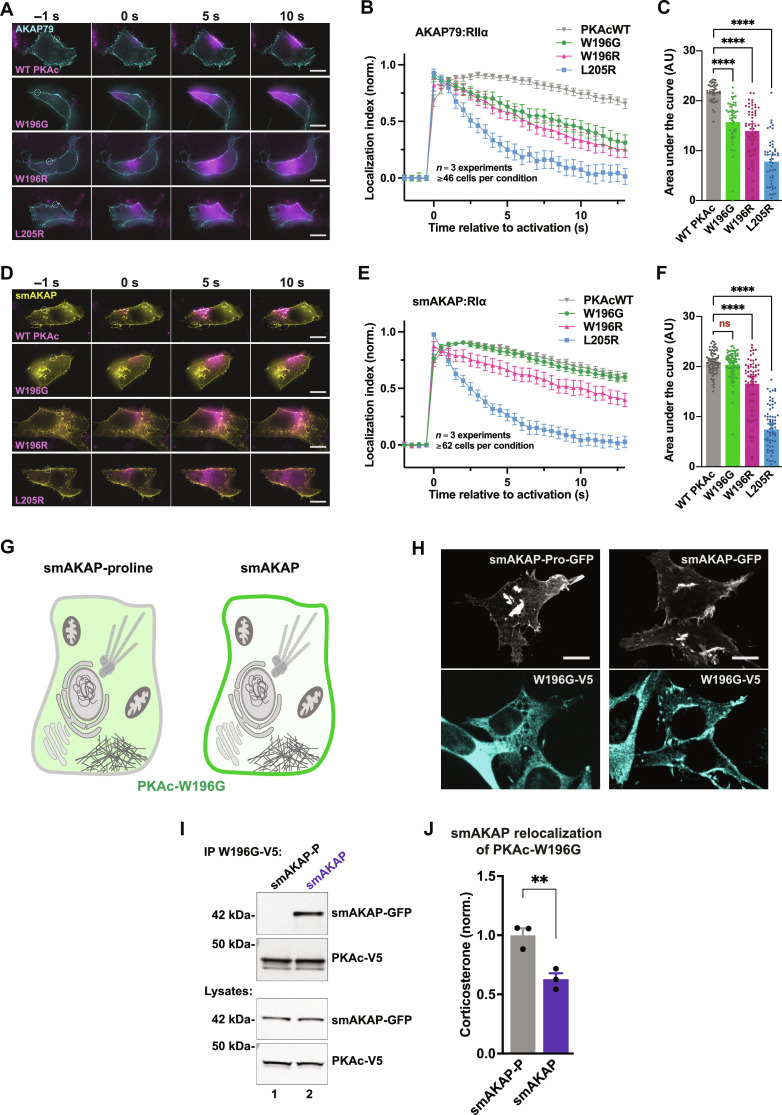
Sequestrating PKAc^W196G^ corrects cortisol overproduction. (**A** to **F**) Type II (A) and type I (D) photoactivation time courses in H295R cells. PKAc variants tagged with photoactivatable mCherry were expressed along with either AKAP79-YFP and RIIα-iRFP [(A) to (C)] or small membrane-bound AKAP (smAKAP)–green fluorescent protein (GFP) and RIα-iRFP [(D) to (F)]. Plotting PKAc localization after photoactivation [(B) and (E)] and area under the curve [(C) and (F)] demonstrates differences among mutants. Localization index = [(intensity of activated region − background intensity)/(intensity of cytosolic region 6 to 8 μm distal − background intensity)]. Scale bars, 10 μm; means ± SE; *n* = 3 replicates with a total of at least 46 [(A) to (C)] and 62 [(D) to (F)] cells per condition; ****P ≤ 0.0001, one-way ANOVA with Dunnett’s correction. ns, not significant. (**G**) Cartoon depiction of experimental design for the PKA anchoring defective smAKAP-proline mutant (left) and smAKAP (right) sequestration experiments. Green demarks expected localization of PKAc-W196G. (**H**) Fluorescent images of ATC7L cells stably expressing PKAc-W196G-V5 (cyan) along with GFP-tagged constructs of either smAKAP-proline (white) or WT smAKAP (white). Scale bars, 10 μm. See also fig. S4. (**I**) Immunoprecipitation of PKAc-W196G from stable W196G/smAKAP-proline (lane 1) or W196G/smAKAP (lane 2) ATC7L adrenal cells. Representative of three biological replicates. (**J**) Corticosterone measurements from stable ATC7L cells coexpressing PKAc^W196G^ with either smAKAP-proline (gray) or smAKAP (purple). Means ± SE; *n* = 3; **P ≤ 0.01, Student’s *t* test.

PKA organization by AKAPs ensures spatial control of second messenger events and contributes to the fidelity of hormone action (*46*–*48*). A fundamental tenet of this anchoring hypothesis is that PKAc action is focused within AKAP signaling islands (*9*, *11*). We therefore used photokinetic imaging to monitor PKAc protein mobility in living cells. Time-course imaging revealed distinctions in PKAc^W196G^ diffusion rates in the vicinity of type II and type I AKAP signaling islands (Fig. 7, A to F, and movies S1 to S4). To interrogate anchored type II complexes, adrenal cells were transfected with membrane-localized AKAP79 and RIIα, as well as PKAc variants tagged with photoactivatable mCherry. Upon photoactivation and 12.5 s of monitoring, 70% of WT PKAc remained at the site of activation (Fig. 7, A, top panels, and B). In contrast, the PKAc^W196G^ mutant and the W196R analog only displayed ~30% residence at the site of photoactivation (Fig. 7, A, middle panels, and B). As expected, the PKAc^L205R^ variant diffused rapidly throughout the cytosol (Fig. 7, A and B). Quantification of the area under the curve from multiple experiments (*n* = 3; ≥45 individual cells per condition) confirmed these results (Fig. 7C). Type I complexes were tested using the RI-selective, small membrane-bound AKAP (smAKAP) along with RIα. These experiments revealed retention of PKAc^W196G^ at the site of photoactivation to the same extent as WT PKAc (Fig. 7D, top two panels). After 12.5 s, 65% of these two variants remained at their sites of activation (Fig. 7, D, top two panels, and E, gray and green traces). PKAc^W196R^ demonstrated an increase in residence with RI (40%) compared to that with RII, and PKAc^L205R^ once again showed total loss of localization (Fig. 7, D, bottom two panels, and E, pink and blue traces). Quantification of the area under the curve details these comparisons (Fig. 7F; *n* = 3; ≥62 individual cells per condition).

The final phase of our study evaluated stress hormone release upon membrane anchoring of PKAc^W196G^. We created ATC7L adrenal cell lines with stable expression of V5-tagged PKAc^W196G^ (fig. S4, A to C). Doxycycline-inducible expression of the RI-selective smAKAP was introduced to target this Cushing’s variant to the plasma membrane (Fig. 7G, right; and fig. S4, D and E). A PKA-anchoring deficient version (smAKAP-proline) was generated to serve as a negative control (Fig. 7G, left; and fig. S4, D and E). Immunofluorescence detection of smAKAP–green fluorescent protein (GFP) demonstrated membrane targeting of both anchoring protein forms (Fig. 7H, top panels, and fig. S4F). Staining for V5-PKAc^W196G^ revealed a diffuse cytoplasmic pattern in smAKAP-proline cells, verifying the lack of recruitment in the control condition (Fig. 7H, left panels). In contrast, V5-PKAc^W196G^ codistributed with smAKAP at the plasma membrane in cells expressing the functional anchoring protein (Fig. 7H, right panels). Coimmunoprecipitation experiments confirmed that PKAc^W196G^ only binds smAKAP (Fig. 7I, top panel, lane 2). Last, corticosterone measurements revealed a 40% reduction in stress hormone production from cells with anchored PKAc^W196G^ compared to smAKAP-proline controls. This finding strongly suggests that cytosolic localization of the Cushing’s syndrome mutant PKAc-W196G is crucial for its pathogenicity and that its redistribution via association with smAKAP can attenuate stress hormone release.

## DISCUSSION

Investigating mutations in the PKA pathway genetically linked to adrenal Cushing’s syndrome has advanced understanding of this endocrine disorder (*19*, *20*, *49*). Here, we use an innovative precision medicine screening protocol and next-generation sequencing to discover a PKAc variant (W196G) present in ~20% of tested patients with adrenal Cushing's syndrome from the Pacific Northwest region of the USA. PKAc^W196G^ exhibits a modified subcellular distribution and a distinct mechanism of action compared to the disease-prevalent PKAc^L205R^ mutant. Both W196G and L205R point mutations occur within a mutational hot spot in exon 7 of the *PRKACA* gene (*25*, *44*, *50*). Scrutiny of The Cancer Genome Atlas, DisGeNET, and ProKinO databases reveals greater mutational diversity at position 196 than any other residue in PKAc (*34*, *51*–*54*). For example, low-frequency somatic mutations that convert this key tryptophan residue into leucine (W196L), serine (W196S), or cysteine (W196C) are detected in several cancers (*34*, *52*, *53*). PKAc^W196G^ and PKAc^W196R^ are the only Cushing’s variants at this position identified to date. Moreover, our primary exome sequencing implies that the frequency of PKAc^W196G^ is likely higher than W196R. However, a comprehensive genomic analysis of different patient cohorts from different geographical regions will be necessary to confirm prevalence. By analogy, full validation of PKAc^L025R^ required exome sequencing of adrenal tissue from five independent patient cohorts on three continents (*24*). Health Insurance Portability and Accountability Act (HIPAA) privacy rules preclude disclosure of clinical phenotypes for the patients harboring a single allele of PKAc^W196G^. Hence, we are unable to trace the unique impact of this pathological kinase variant within the broader adrenal Cushing’s syndrome community. It is worth speculating that the increased catalytic efficiency of PKAc^W196G^ may enhance phosphorylation of substrates as compared to the more prevalent PKAc^L205R^.

Patients harboring PKAc^W196G^ have elevated RIα levels, implying that cAMP responses are skewed toward local signaling through the type I kinase (Fig. 6G). This undoubtedly sensitizes adenomas to cAMP as the type I PKA holoenzyme is activated at sevenfold lower levels of second messenger (*43*–*45*). Biased PKA isotype signaling has been observed in both benign and malignant neoplasms, although the outcomes are pleiotropic and context-dependent (*55*, *56*). For example, germline mutations in Carney Complex that ablate RIα predispose patients to myxomas and thyroid and gonadal tumors (*57*). Sporadic RIα loss is also recurrent in adrenocortical carcinoma (*58*). Conversely, stabilization of RI subunits is evident in the adolescent liver cancer fibrolamellar carcinoma (*59*). Collectively, these clinical observations argue that up-regulation of RIα may be a protective mechanism to regain spatiotemporal control of PKAc activity. This theory is supported by the rescue experiments presented in Fig. 6I showing that excess RIIα does not affect corticosterone release from PKAc^W196G^-expressing adrenal cells, whereas overexpression of RIα returns stress hormone release to baseline levels. It is now unclear whether RI subunits fulfill similar protective roles across the full range of Cushing’s syndrome, including ACTH-producing pituitary adenomas and familial micronodular adrenocortical dysplasia (*17*, *18*, *26*, *60*).

A defining feature of PKAc^W196G^ compared to other known Cushing’s mutations is its preferential association with RI. Cell-based studies presented in Fig. 6 (A to D) show that PKAc^W196G^ displays a higher affinity than the W196R analog for RIα and the dual-function AKAP220. This is consistent with biochemical evidence showing that introduction of arginine at position 196 decreases RI affinity nearly sixfold (*61*). Structural analysis identifies tryptophan-196 as a critical residue located in the activation loop of the kinase that interfaces with PKA regulatory subunits (*62*). However, how disease-causing mutations at position 196 affect PKA holoenzyme formation and regulation are not fully understood (*29*, *59*, *63*). Dynamic simulation studies presented in Fig. 4 provide a compelling mechanistic explanation. Replacing the large indole ring of tryptophan-196 with glycine creates a unique cleft between the associated PKA subunits. This cavity occupies 394 Å^3^ at the RIα:C interface but increases to 653 Å^3^ at the RIIα:C interface. The extended distance between side chains causes a packing defect that impairs RII autoinhibition of the catalytic subunit, which is readily recapitulated biochemically (Figs. 5 and 6). Primary sequence differences between RI and RII help explain these topographical dissimilarities between isotypes. In RIα, the flexible methionine-234 side chain has sufficient range of motion to interact with PKAc^W196G^. In contrast, a smaller valine-239 side chain at the corresponding position in RIIα is less effective in reaching across the cleft, resulting in weaker affinity for PKAc^W196G^. Thus, seemingly trivial conformational perturbations in the kinase domain can profoundly influence the R subunit selectivity of this Cushing’s variant.

The W196G mutation creates a single–amino acid substitution in the activation loop of the kinase, which is a hotspot for PKA regulation (*64*, *65*). However, enzyme activity measurements presented in Figs. 2 and 5 show that the W196G mutant and WT PKAc display similar kinetic parameters and sensitivity to the heat-stable inhibitor PKI. This is a somewhat unexpected finding as somatic mutations in the activation loop of most kinases alter the catalytic activity of the enzyme and can promote resistance to clinical inhibitors (*66*–*68*). Unlike most kinases and other Cushing’s variants, the mechanics of the phosphotransferase reaction are preserved in PKAc^W196G^ (*26*, *28*). Rather, our study shows that the W196G mutation biases PKAc interactions toward a subset of cellular protein partners. For example, photokinetic imaging experiments in Fig. 7 demonstrate that PKAc^W196G^ is preferentially retained within smAKAP signaling islands through association with RIα. Further, sequestering this Cushing’s variant at the cell’s periphery reduces stress hormone release. Possible explanations for this functional rescue include removal of PKAc^W196G^ from mitochondria where the mutant kinase could access the steroidogenic acute regulatory protein (StAR) and 11β-hydroxylase, the rate-limiting and terminal enzymes in cortisol biosynthesis, respectively (*25*, *69*, *70*). Alternatively, concentrating PKA phosphorylation events at the plasma membrane may reduce access to transcription factors governing expression of StAR and other cortisol production machinery (*71*). Regardless, these results suggest that where and when PKAc^W196G^ catalyzes protein phosphorylation directly affects cortisol production and the pathological impact of this Cushing’s kinase.

Unlike other Cushing’s variants, subcellular targeting of PKAc^W196G^ is principally governed though its preferential association with type I R subunits and dual-function AKAPs. This alters subcellular localization of the kinase, as reflected in our proximity proteomic data, where PKAc^W196G^ displays enhanced proximity to actin filaments and mitochondrial proteins (Fig. 3I). Local PKA phosphorylation events at mitochondria could have a direct impact on cortisol production via StAR (*72*–*74*). Furthermore, imaging of Cushing’s patient tissue in Fig. 2 shows that PKA^W196G^ diffusely populates the cytoplasm of adenomas. This contrasts with the WT kinase, which is precisely organized at the periphery of normal adrenal cells. Dispersed distribution of PKAc^W196G^ may be a result of a dynamic equilibrium between the anchored and untethered pools of this Cushing’s kinase, because RI generally binds its anchoring proteins at lower affinities than RII (*39*, *40*, *69*, *75*, *76*). Irrespective of the precise mechanisms at play, compartmentalization of PKAc^W196G^ is distorted in a manner that either increases off-target phosphorylation events or constrains this Cushing’s kinase in proximity to substrates that potentiate disease. Overall, this work identifies an unexpected pathophysiology of adrenal Cushing’s syndrome and highlights the importance of spatial biology and kinase anchoring as nascent facets of molecular medicine.

## MATERIALS AND METHODS

### Mice

The PKA-CαR (W196R conditional knock-in) mice were provided by S. McKnight (University of Washington) and gifted to us by D. Pattabiraman (Dartmouth) (*77*). These mice carry a *PRKACA* allele encoding the W196R variant in a Cre-dependent manner. To make adrenal cortex–specific mutant mice, we crossed these to aldosterone synthase (AS)–Cre mice, which were a gift from D. Breault (*78*). Adult (11- to 13-month) mice of both sexes were used for our studies. PKA-CαR mice were maintained on a C57BL/6J background. AS-Cre mice were backcrossed to C57BL/6J a minimum of three times, and all experiments used randomly assigned littermates as controls. Control mice included fully WT and singly heterozygous littermates for either floxed W196R or AS-Cre. Mice were housed in groups of 2 or more with littermates of the same sex until the night before the procedure. To minimize stress responses, mice were singly housed the night before the procedure and cages were left on racks until euthanasia. On the day of the procedure, mice were quickly moved to a separate room and immediately euthanized by isoflurane and cervical dislocation. Adrenal glands were dissected from acutely euthanized mice and frozen on dry ice for immunoblot analysis. All mice were cared for and euthanized in accordance with University of Washington regulatory standards and approved University of Washington Institutional Animal Care and Use Committee protocols.

### Human tissue

Formalin-fixed, paraffin-embedded (FFPE) human adrenal cortical adenoma samples were obtained from the University of Washington Northwest BioSpecimen resource. Adherence to ethical standards was ensured by total de-identification before use in research. Samples were deparaffinized by placing slides in 100% xylenes once for 10 min and once for 5 min. Samples were then rehydrated by placing slides in 100% ethanol twice for 10 min each, followed by 95% ethanol for 10 min and 80% ethanol for 10 min, and deionized water two times for 5 min each. Specific human tissue assays are described in further detail below.

### Cell lines

All mammalian cells were grown under 37°C, 5% CO_2_ incubation conditions. Female NCI-H295R cells were purchased from American Type Culture Collection (ATCC) in September 2019 (CVCL_0458) and were maintained in ATCC Dulbecco’s modified Eagle’s medium (DMEM):F12 medium containing 2.5% Corning NuSerum I and 1% Corning ITS+ supplement. Male ATC7L cells were maintained in Gibco DMEM:F12 with added 5 ml l-glutamine (200 mM) and 5 ml of Gibco ITS supplement (*79*). Virus was produced in female HEK-293T cells purchased from Dharmacon in 2015 and grown in DMEM (Gibco) medium with 10% fetal bovine serum (HyClone). HEK-293T cells for expression and purification of affinity-purified recombinant PKA proteins were cultured in DMEM (Lonza) supplemented with 10% fetal bovine serum (HyClone), penicillin (50 U/ml), and streptomycin (0.25 μg/ml; Lonza).

### Microbe strains

All purified proteins were produced in BL21 (DE3) pLysS *Escherichia coli* cells (Novagen) and expression induced with 0.5 mM isopropyl-β-D-thiogalactopyranoside (IPTG) for 18 hours at 18°C. Amplification of nonviral mammalian expression plasmids was performed in GC10 competent cells (Genesee) and grown at 37°C. Amplification of viral vectors was performed in either Stbl3 (Invitrogen) or NEB Stable (New England Biolabs) competent cells and grown at 30°C.

### Human tissue sequencing

FFPE sections were deparaffinized and rehydrated in a series of xylenes and decreasing ethanol concentrations, as described above, and scraped from slides into 1.5-ml tubes. DNA was extracted by incubating in 200 μl of a mixture containing 191 μl of Gitshier buffer, 2 μl of 2-mercaptoethanol, and 7 μl of proteinase K overnight at 55°C. Samples were then heat-inactivated and centrifuged at 10,000*g* for 5 min. Last, 1 μl of the supernatant was used as template for amplification of *PRKACA* exons 6 to 8. PCR was performed with the following conditions and primer sequences: 36.2 μl of ddH_2_O, 10 μl of 5× HF Phusion buffer (NEB), 1 μl of 10 mM mixed deoxynucleotide triphosphates (dNTPs), 1.5 μl of combined primers at 10 μM each, and 0.3 μl of Hot-Start Phusion polymerase (NEB) in a Bio-Rad thermocycler; 94°C for 1 min, a repeating sequence of 94°C for 20 s, 60°C for 30 s, 72°C for 1 min (repeat 30×), and a final elongation at 72°C for 5 min. Forward primer: CCTCCCCATTTGTCCCCATC. Reverse primer: CTTCCCAGAGACGATCTTCTCATAG. Next-generation sequencing was performed on whole-mounted sections by Genewiz according to the standard company procedures.

### Structural models

Model of PKA catalytic subunit in Fig. 1D was made using PyMOL database file 6E99.

### Antibodies

The following antibodies were used in our studies: AKAP220, custom rabbit antibody [Western blot (WB)]; AKAP79, custom V089 (WB); GFP, Rockland, 600-101-215 [immunofluorescence (IF), immunoprecipitation (IP), and WB]; HA-HRP, Roche, 12013819001 (WB); NeutrAvidin-HRP, Pierce, 31030 (WB); PKAc, BD Biosciences, 610981 (IF and WB); PKAc, Cell Signaling Technology, 5842 (IP and WB); PKA RIα, Cell Signaling Technology, 5675 (WB); PKA RIIα, BD Biosciences, 612243 (WB); PKA RIIβ, BD Biosciences, 610626; V5-HRP, Invitrogen, 46-0708 (WB); and V5-tag, Thermo Fisher Scientific, R96025 (IF and IP).

### Plasmid generation

Standard cloning was performed using PCR [36.2 μl of ddH_2_O, 10 μl of 5× HF Phusion buffer (NEB), 1 μl of 10 mM mixed dNTPs, 1.5 μl of combined primers at 10 μM each, 1 μl of template DNA at 5 ng/μl, and 0.3 μl of Hot-Start Phusion polymerase (NEB)] in a Bio-Rad thermocycler. Thermocycling protocols varied depending on primer conditions and length of target region. For mutagenesis protocols, Dpn I restriction enzyme and polynucleotide kinase were used (NEB). Some constructs were made using Gateway cloning system (Thermo Fisher Scientific). Ligation was performed with T4 DNA ligase (NEB) for 10 to 20 min at room temperature (RT) using the manufacturer’s recommendations. Transformation into competent bacteria (see the “Microbe strains” section) was performed on ice for 15 to 30 min before heat shock for 30 s at 37°C.

### Immunoprecipitations

Cell lysates were made using lysis buffer containing 0.5 to 1% Triton X-100, 130 mM NaCl, 20 mM NaF, 2 mM EDTA, and 50 mM tris (pH 7.5) (at 4°C) along with 1 mM 4-(2-aminoethyl)-benzenesulfonyl fluoride (AEBSF), 10 μM leupeptin, and 1 mM benzamidine. Lysates were incubated 5 min on ice and spun at 15,000*g* for 8 min at 4°C. Protein concentration was measured by BCA (Thermo Fisher Scientific) and adjusted to either 0.5 or 1 mg/ml using lysis buffer. Samples (300 to 500 μl) were precleared by rotating with 20 μl of protein G agarose (50% slurry) for 30 min at 4°C. Supernatants were then incubated with 1 to 2 μg of the appropriate antibody overnight. In the morning, 25 μl of protein G agarose (50% slurry) was added, and samples were returned to rotation for 1 hour. Beads were washed with lysis buffer, centrifuged at 5000*g* three times, and then aspirated with a 27-gauge needle before resuspending in 1× polyacrylamide gel electrophoresis (PAGE) sample buffer (3% β-mercaptoethanol, final) and heating at 80°C for 10 min. Figures are representatives for at least three experimental replicates.

### Immunoblotting

Human tissue for immunoblotting was processed as described above, followed by use of a Qproteome FFPE Tissue kit (QIAGEN, 37623). Cell lysates were made using radioimmunoprecipitation assay (RIPA) lysis buffer [1% NP-40 Tergitol, 0.5% deoxycholate, 0.1% SDS, 130 mM NaCl, 20 mM NaF, 2 mM EDTA, and 20 mM tris (pH 7.5) (at 4°C) along with 1 mM AEBSF, 10 μM leupeptin, 1 mM benzamidine, and 10 mM β-glycerophosphate]. Mouse adrenal protein extracts were made by homogenizing whole glands in RIPA buffer. Samples were incubated 5 min on ice and spun at 15,000*g* for 8 min at 4°C. Protein concentration was measured by BCA (Thermo Fisher Scientific). Gels were loaded with 10 to 30 μg of protein after heating for 10 min at 80°C with PAGE sample buffer containing 3% (final) β-mercaptoethanol. Proteins were transferred to nitrocellulose or polyvinylidene difluoride, incubated with Ponceau S stain to measure total protein loading, blocked in 5% milk tris-buffered saline with Tween 20 (TBST) for 30 min at RT, and probed with antibodies in 5% bovine serum albumin (BSA) TBST overnight at 4°C. Membranes were washed three times in TBST and then incubated with secondary antibodies conjugated to HRP diluted in 5% milk TBST for 1 hour at RT. After washing again three times in TBST, signals were visualized with SuperSignal West Pico Chemiluminescent Substrate (Thermo Fisher Scientific) on the Invitrogen iBright Imaging System. Quantification was performed with ImageJ analysis software by measuring signal minus background for each band and dividing by the appropriate control signal, as indicated in each figure.

### Hormone measurements

Cells were washed once in phosphate-buffered saline (PBS), and medium was changed 75 min before harvest. Harvested medium samples were snap-frozen until assayed. To assay, samples were diluted and subjected to measurement using Cayman Cortisol or Corticosterone ELISA kits. Values were determined using an absorbance plate reader and subsequent curve fit analysis. All experiments were completed in at least three separate biological replicates. Measurements were normalized to control level(s) within each replicate.

### Modeling and MD simulations

The initial structure for the simulations was obtained from the co-crystal structure of the catalytic subunit of PKA (PKAc) with RIα (Protein Data Bank ID: 3PVB). W196G and W196R mutations were made in silico in PyMOL version 2.3.5 through the mutagenesis tool. The rotameric conformation with the least steric clash was selected. The resulting mutant structure for W196G served as a template to model PKAc bound to RIIα using the SWISS-MODEL server. The cavities at the interface for the different structures were calculated using Fpocket tool using the Monte Carlo method. Three independent 500-ns all-atom MD simulations were performed using GROMACS 2020 software for the RIα:CW196G and RIIα:CW196G complexes. The CHARMM36-March2019 force field was applied to parameterize the system, and the protein complex was solvated using the transferable intermolecular potential with 3 points (TIP3P) water model within a dodecahedron box. To neutralize the protein charge, sodium, and chloride ions were added. Nonbonded interactions were defined using the Verlet cutoff method, and long-range interactions were calculated using the particle mesh Ewald approach. The energy minimization process involved using the steepest-descent algorithm followed by the conjugate descent, with maximum force (*F*_max_) less than 200 kJ mol^−1^ nm^−1^. The canonical ensemble was carried out by heating the system from 0 to 310 K, using velocity rescaling for 100 ps. The isothermal-isobaric ensemble (*P* = 1 bar, *T* = 310 K) was carried out for 100 ps using the Berendsen barostat. After the isothermal-isobaric ensemble, unrestrained MD productions were collected with a time step of 2 fs. The resulting trajectories were processed and analyzed using the built-in tools of GROMACS. Structural visualization was performed using PyMOL version 2.3.5. In addition, the contacts between PKAc and the regulatory subunits during the MD simulations were quantified using PyContact version 1.0.4.

### Photoactivation microscopy

NCI-H295R cells were grown in glass bottom dishes and transfected using Lipofectamine 3000 48 hours before imaging. Mammalian expression plasmids with cytomegalovirus promoters and encoding AKAP79-YFP, smAKAP-GFP, RIIα-iRFP, RIα-iRFP, and either WT, W196G, L205R, or W196R PKAc tagged with photoactivatable mCherry were used. Imaging was performed using a GE OMX SR system. All four conditions (PKAc variants) within each experimental replicate were assayed same day. Exposure and laser intensity were optimized for each experimental replicate and held constant among experimental conditions. Photoactivation laser duration was kept under 50 ms to activate a discrete area with minimal spread in the first image collected after activation. Images were collected at 2 Hz in three channels. A baseline of four images was taken before activation of the PKAc fluorophore. Cells were selected for imaging only when R-iRFP signal was colocalized with the AKAP. Secondary screening for this was performed post hoc. Time courses were measured using ImageJ analysis software (Fiji). A localization index [(intensity of the activated region minus background)/(intensity of cytosolic region 6 to 8 μm distal − background)] was used to interrogate change in fluorescent signal localization over time (mobility). For representative images, deconvolution, and alignment were performed using OMX software, and Fiji was used for further image processing.

### Proximity biotinylation and sample preparation for MS

Stable NCI-H295R adrenal cell lines were made using lentivirus encoding a tetracycline-responsive promoter and variants of PKAc tagged with V5 and miniTurbo biotin ligase at the C terminus (*31*). Induction with doxycycline (0.5 to 1 μg/ml) was optimized to yield a subtle overexpression of the bait constructs at 20% of endogenous PKAc levels, as determined by PKAc immunoblotting and quantification using ImageJ. miniTurbo-tagged variant expression was induced for 48 hours before application of 50 μM biotin in dimethyl sulfoxide. Cells were incubated for 1 hour at 37°C, washed two times for 1 min using 10 ml of PBS to deplete excess biotin, and then lysed using RIPA buffer (as described above). Protein concentrations were measured by BCA, and samples were diluted to 1 ml of RIPA buffer (0.5 mg/ml) and placed in low protein binding tubes (Thermo Fisher Scientific) containing 25 μl of Nanolink magnetic streptavidin beads. Tubes were rotated 1 hour at RT and placed on a magnet. Supernatant was saved for diagnostics, and samples were washed in RIPA 2×, 2 M urea in 20 mM tris 2×, and 25 mM tris 2×. Samples were resuspended in 8 M urea in 100 mM tris (pH 8.5) with 5 mM tris(2-carboxyethyl)phosphine hydrochloride and 10 mM chloroacetamide and then incubated at 37°C for 1 hour. For digestion, samples were diluted twofold with 100 mM tetraethylammonium bromide (TEAB), and 1 μg of LysC was added before incubation for 2 hours shaking at 37°C. Samples were again diluted with 100 mM TEAB, and 1 μg of Trypsin was added before incubation overnight shaking at 37°C. In the morning, samples were acidified to 1% formic acid and loaded on C18 StageTips.

### LC-MS/MS analysis

Peptides were eluted from StageTips using 40% (v/v) acetonitrile, 1% (v/v) FA in water, and then loaded on a self-pulled 360-μm–outer diameter–by–100-μm–inside diameter 20-cm column with a 7-μm tip packed with 3 μm of Reprosil C18 resin (Dr. Maisch, Germany). Analysis was performed by nanoLC-MS in a 90-min gradient from 15 to 38% mobile phase B at 300 nl/min using a Thermo EASY nLC 1200 system (mobile phase A, 0.1% acetic acid; mobile phase B, 0.1% acetic acid and 80% acetonitrile). Mass spectra were collected from an Orbitrap Fusion Lumos Tribrid Mass Spectrometer using the following settings: for MS1, Orbitrap FTMS [*R* = 60 k at 200 mass/charge ratio (*m*/*z*); *m*/*z* 350 to 1600; 7 × 10^5^ target; max 20-ms ion injection time); for MS2, Top Speed data-dependent acquisition with 3-s cycle time was used, higher-energy C-trap dissociation (HCD) MS2 spectra were collected using the Orbitrap mass analyzer [*R* = 30 k at 200 *m*/*z*; 31% collision energy (CE); 5 × 10^4^ target; max 100-ms injection time], and an intensity filter was set at 2.5 × 10^4^ and dynamic exclusion for 45 s. Mass spectra were searched against the UniProt human reference proteome downloaded on 6 July 2016 using MaxQuant v1.6.2.6. Detailed MaxQuant settings: “Label-free quantification” was turned on but not “match between run,” and no fractionation was set; Trypsin/P was selected in digestion setting. Other settings were kept as default. Protein network prediction and gene ontology analysis were performed using STRING database version 11.5, and gene ontology enrichment analysis was performed using The Gene Ontology Resource powered by PANTHER.

### Recombinant protein purification

WT, W196G, W196R, and L205R PKAc subunit; RIα and RIIα regulatory subunits; and PKI were produced in BL21 (DE3) pLysS *E. coli* cells (Novagen), and expression was induced with 0.5 mM IPTG for 18 hours at 18°C. Proteins were purified as N-terminal His6-tag fusions by immobilized metal affinity chromatography followed by size exclusion chromatography using a HiLoad 16/600 Superdex 200 column (GE Healthcare) equilibrated in 50 mM tris-HCl (pH 7.4), 100 mM NaCl, 1 mM dithiothreitol (DTT), and 10% (v/v) glycerol. PKAc variants (including catalytically inactive K72H PKA) expressing an N-terminal 3C protease cleavable MYC tag were purified from transfected HEK-293T cells. HEK-293T cells were transfected using a 3:1 polyethylenimine (average *M*_w_, ~25,000 Da; Sigma-Aldrich) to total DNA ratio (10 μg of PKA plasmid) in a single–10-cm tissue culture plate (five plates were used per protein). Forty-eight hours after transfection, cells were resuspended in ice-cold lysis buffer [50 mM tris-HCl (pH 7.5), 150 mM NaCl, 10% (v/v) glycerol, and 1% (v/v) Triton X-100 and supplemented with protease and phosphatase inhibitor tablets (Roche)] and disrupted with brief sonication. Lysates were clarified by centrifugation at 20 817*g* for 20 min at 4°C, and recombinant proteins were immune-precipitated using MYC-agarose beads and eluted from the beads using 3C protease before assay.

### Protein kinase assays

PKA kinase assays using recombinant proteins extracted from human and bacterial cells were performed using a real-time mobility shift-based microfluidic system, as described previously (*80*), in the presence of 2 μM of the fluorescent-tagged Kemptide substrate (5-FAM-LRRASLG-CONH_2_) and 1 mM ATP (as standard). Pressure and voltage settings were −1.8 psi and −2250 V (upstream voltage) and −500 V (downstream voltage), respectively. All assays were performed in 50 mM Hepes (pH 7.4), 0.015% (v/v) Brij-35, 1 mM DTT, and 5 mM MgCl_2_, and peptide phosphorylation was detected in real time as the ratio of phosphopeptide:peptide in each sample of a 384-well plate. Changes in PKA activity in the presence of regulatory proteins (PKI, RIα, and RIIα, assayed at the indicated concentrations) were quantified as the rate of phosphate incorporation into the substrate peptide (pmol phosphate min^−1^ μM enzyme^−1^) and then normalized with respect to control assays (lacking regulatory proteins). To prevent ATP depletion and consequential loss of assay linearity, phosphate incorporation into the peptide was generally limited to <20%. ATP *K*_m_ values were determined by nonlinear regression analysis using GraphPad Prism software. Unless otherwise specified, PKA WT and W196 mutants were assayed at a final concentration of 0.3 nM, and L205R and K72H mutants were assayed at 6 μM to account for their lower rates of activity toward Kemptide.

### Differential scanning fluorimetry

Thermal-shift assays were performed using a StepOnePlus Real-Time PCR machine (Life Technologies) using Sypro-Orange dye (Invitrogen) and thermal ramping (0.3°C in step intervals between 25° and 94°C). PKA proteins were diluted to a final concentration of 5 μM in 50 mM tris-HCl (pH 7.4) and 100 mM NaCl in the presence or absence of 1 mM ATP (and 10 mM MgCl_2_) or 10 μM PKI with ATP-MgCl_2_ and were assayed as described previously (*80*). Normalized data were processed using the Boltzmann equation to generate sigmoidal denaturation curves, and average (*T*_m_/∆*T*_m_) values calculated using GraphPad Prism software.

### PKAc autophosphorylation MS

Concentrated purified PKA (10 μg) was diluted to 400 μl in 100 mM ammonium bicarbonate (pH 8.0; ~60-fold dilution) before being subject to reduction (with dithiothreitol) and alkylation (with iodoacetamide) as previously described (*80*). The eluent was digested with 33:1 (w/w) trypsin gold (Promega) at 37°C for 18 hours with 600 rpm shaking. Digests were then subject to strong cation exchange using in-house packed stage tip cleanup, as previously described. Dried peptides were solubilized in 20 μl of 3% (v/v) acetonitrile and 0.1% (v/v) trifluoroacetic acid (TFA) in water, sonicated for 10 min, and centrifuged at 13,000*g* for 15 min at 4°C before separation and analysis by LC-MS/MS using an UltiMate 3000 nano system (Dionex), over a 60-min gradient, as described in (*80*). Briefly, samples were loaded at a rate of 12 μl/min onto a trapping column (PepMap100, C18, 300 μm by 5 mm) in loading solution [3% (v/v) acetonitrile, 0.1% (v/v) TFA] before being resolved on an analytical column (Easy-Spray C18 75 μm by 500 mm, 2-μm bead diameter column) using a gradient of 97% A [0.1% (v/v) formic acid]:3% B [80% (v/v) acetonitrile and 0.1% (v/v) formic acid] to 80% B over 30 min at a flow rate of 300 nl/min. All data acquisition was performed using a Thermo Orbitrap Fusion Tribrid mass spectrometer (Thermo Fisher Scientific), with HCD fragmentation set at 32% normalized collision energy for 2+ to 5+ charge states using a 3-s cycle time. MS1 spectra were acquired in the Orbitrap (120 K resolution at 200 *m*/*z*) over a range of 350 to 1400 *m*/*z*, AGC target = standard, maximum injection time = auto, with an intensity threshold for fragmentation of 2e4. MS2 spectra were acquired in the Orbitrap (30 K resolution at 200 *m*/*z*), AGC target = standard, maximum injection time = dynamic. A dynamic exclusion window of 20 s was applied at a mass tolerance of 10 parts per million (ppm). Data were analyzed by Proteome Discoverer 2.4 using a custom database of the UniProt *E. coli* proteome (updated January 2021) supplemented with the His-tagged mouse PKA protein and relevant mutants, searched with fixed modification = carbamidomethylation (C), variable modifications = oxidation (M) and phospho (S/T/Y), instrument type = electrospray ionization–Fourier transform ion cyclotron resonance, MS1 mass tolerance = 10 ppm, MS2 mass tolerance = 0.01 Da, ptmRS node on, set to a score of >99.0. For label-free relative quantification of phosphopeptide abundance of each PKA variant, the minora feature detector was active and set to calculate the area under the curve for peptide *m*/*z* ions. To account for potential protein loading variability during analysis, the abundance of phosphopeptide ions were normalized against the total protein abundance (determined by the Hi3 method in minora feature detector mode).

### Immunofluorescent staining

For human tissue staining, FFPE sections were deparaffinized and rehydrated as described above. Antigen retrieval was performed by placing slides in a chamber with pre-boiled 10 mM sodium citrate buffer (pH 6.0). The chamber was then placed inside of a vegetable steamer for 1 hour. Slides were placed under cold running water for 10 min before permeabilization in 0.4% Triton X-100/PBS for 7 min. Blocking was carried out in 5% BSA and 10% donkey serum in PBST (containing 0.05% Tween 20) for 2 hours at RT. For immunofluorescence, slides were incubated with primary antibodies in 5% BSA in PBST overnight at 4°C. Cells were washed three times in PBST for 10 min each and incubated with Alexa Fluor–conjugated secondary antibodies and 4′,6-diamidino-2-phenylindole (DAPI) in 3% BSA in PBST for 1 hour at RT. Slides were then washed six times for 10 min each in PBST. Samples were mounted on glass slides using ProLong Diamond antifade mountant (Thermo Fisher Scientific) and cured overnight. Images were acquired using a Leica DMI6000B inverted microscope with a spinning disk confocal head (Yokagawa) and a CoolSnap HQ camera (Photometrics) controlled by MetaMorph 7.6.4 (Molecular Devices). For immunofluorescent staining of cultured cell lines, cells were plated on acid-washed coverslips and induced with doxycycline (1 μg/ml) 48 hours before harvest. Cells were fixed in 4% PFA for 15 min at RT and washed three times in PBS. Coverslips were moved to humidity chamber and blocked for 1 hour at RT in 3% BSA and 0.3% Triton X-100. Primary antibodies were diluted in blocking solution and applied to coverslips overnight at 4°C. Coverslips were washed three times with PBS, incubated with fluorescent secondary antibodies (1:1000) and DAPI (~1:10,000), and washed three times in PBS again before mounting. Images were taken on a Keyence BZ-X710 microscope and processed/analyzed using ImageJ analysis software (Fiji). Line scan analysis was performed on a *z*-section identified as the clearest DAPI signal, indicating focus on the vertical center of the cell. Lines covered the complete length of a cell, crossing the nucleus. Fluorescent intensity was measured for PKAc and DAPI signals on composite images. DAPI values were used to identify nuclear boundaries. Average pixel intensity values for PKAc signal were calculated for nuclear and non-nuclear compartments, and the ratio of nuclear to non-nuclear signal was determined in each cell.

### Quantification and statistical analyses

Data quantification and statistical analyses were performed with GraphPad Prism 9 for Mac. All data are presented as means ± SD or means ± SE, as noted in the figure legend, with individual values displayed when possible. Individual figure legends contain specific information on statistical parameters. Experiments involving more than three conditions used one-way analysis of variance (ANOVA) with subsequent *t* tests corrected for multiple comparisons. Specific statistical approaches were determined on the basis of the experimental design, distributions, and parameters for each dataset.
