# Supplementary material for: Discovery of a Cushing’s syndrome protein kinase A mutant that biases signaling through type I AKAPs
Source: Sci Adv. 2024 Feb 21;10(8):eadl1258. doi: 10.1126/sciadv.adl1258 (PMC10881042; doi:10.1126/sciadv.adl1258)
Supplement: Supplementary file 1 — Figs. S1 to S4 Legends for movies S1 to S4 [file sciadv.adl1258_sm.pdf]

Science Advances
AAAS

Supplementary Materials for

Discovery of a Cushing's syndrome protein kinase A mutant that biases signaling through type I AKAPs

Mitchell H. Omar *et al.*

Corresponding author: John D. Scott, scottjdw@uw.edu; Mitchell H. Omar, momar@unr.edu

Sci. Adv. **10**, eadl1258 (2024)
DOI: 10.1126/sciadv.adl1258

The PDF file includes:

Figs. S1 to S4
Legends for movies S1 to S4

Other Supplementary Material for this manuscript includes the following:

Movies S1 to S4

SUPPLEMENTAL FIGURES

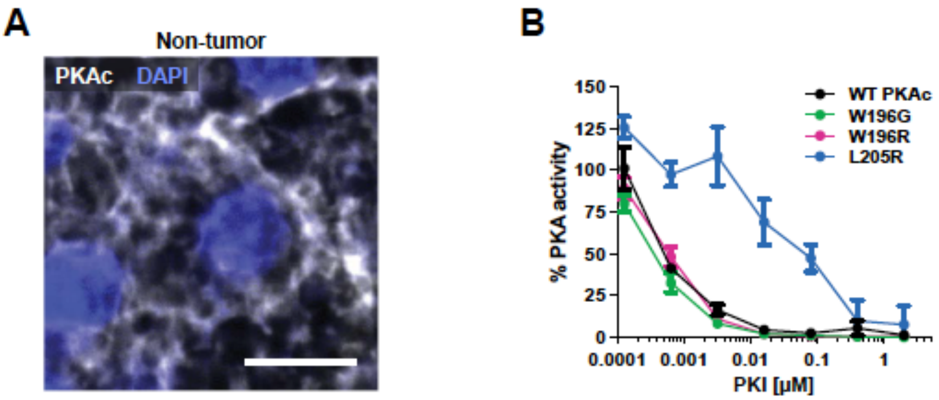

Figure S1: Non-tumor patient tissue and PKI inhibition of Cushing's mutants

(A) High magnification of tumor-adjacent adrenal tissue from a Cushing's syndrome patient stained for PKAc (white) and nuclei (DAPI, blue). Scale bar = 10 µm.

(B) PKAc activity measurements (using Kemptide as a substrate) in the presence of the PKI_{5-24} inhibitory peptide. Percent (%) kinase activity is monitored against concentration (µM) of PKI_{5-24} inhibitory peptide. Mean ± SD; n=4 independent replicates.

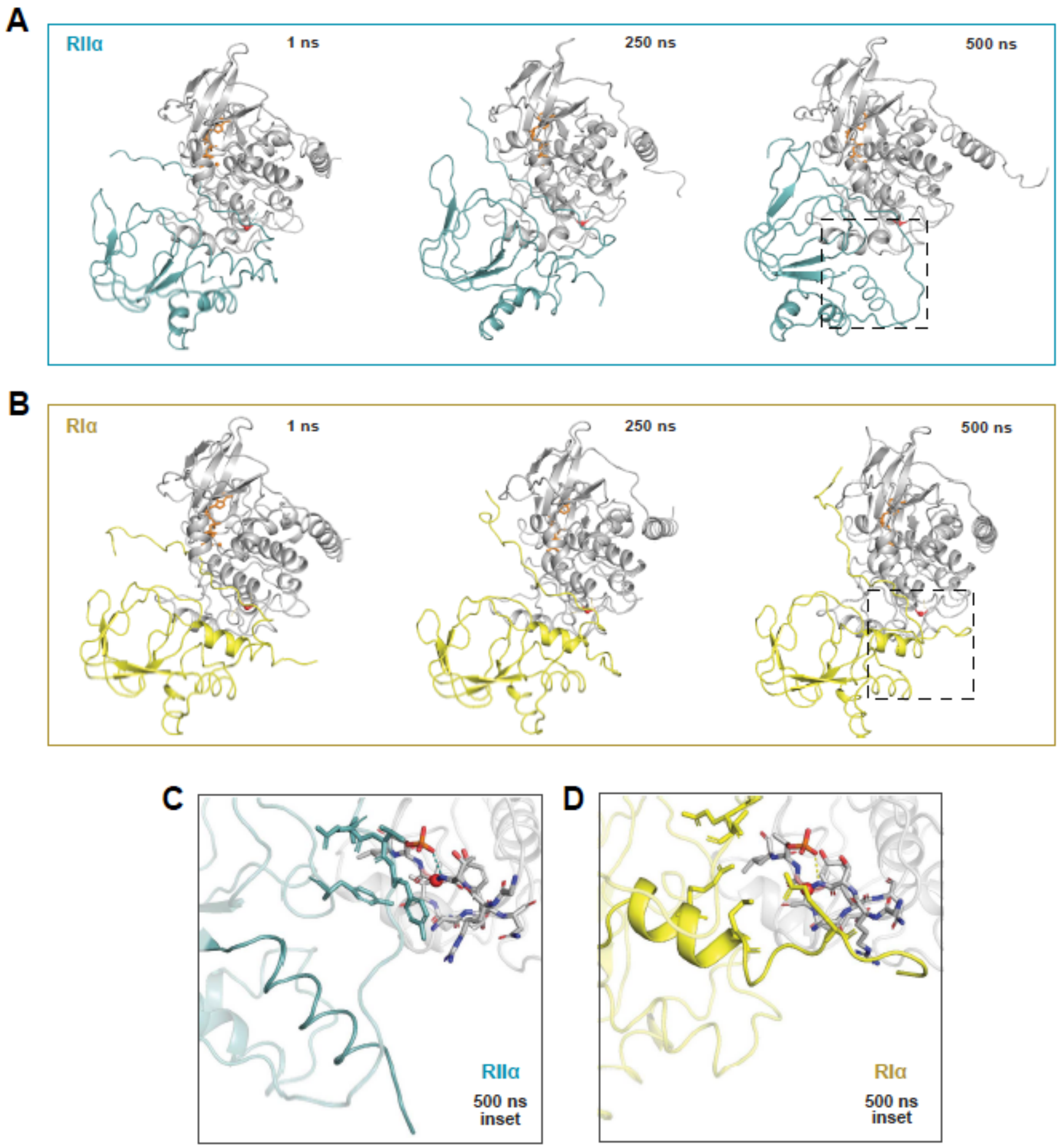

Figure S2: In silico R:C structural analyses

(A-B) Expanded and more detailed representations of the molecular simulation timecourses presented in figures 4D and 4E. Molecular dynamic timecourses of PKAc^{W196G} (gray) in complex with (A) RII (cyan) and (B) RI (yellow). (C-D) Magnification of dashed box in S2A and S2B.

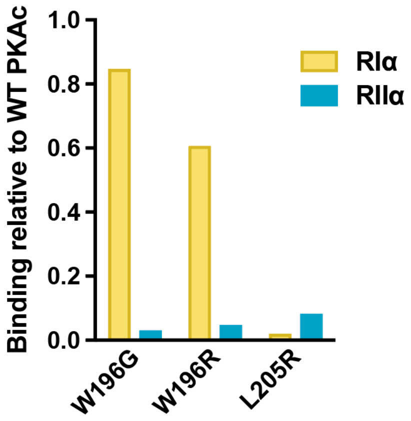

Figure S3: HEK293-immunoprecipitated W196G and W196R prefer RI

Quantification of RIα (yellow) and RIIα (teal) PKA regulatory subunits co-precipitated from HEK293 cells by immunoprecipitated PKAc variants, as determined by LC-MS. Data presented as amount bound relative to WT PKAc.

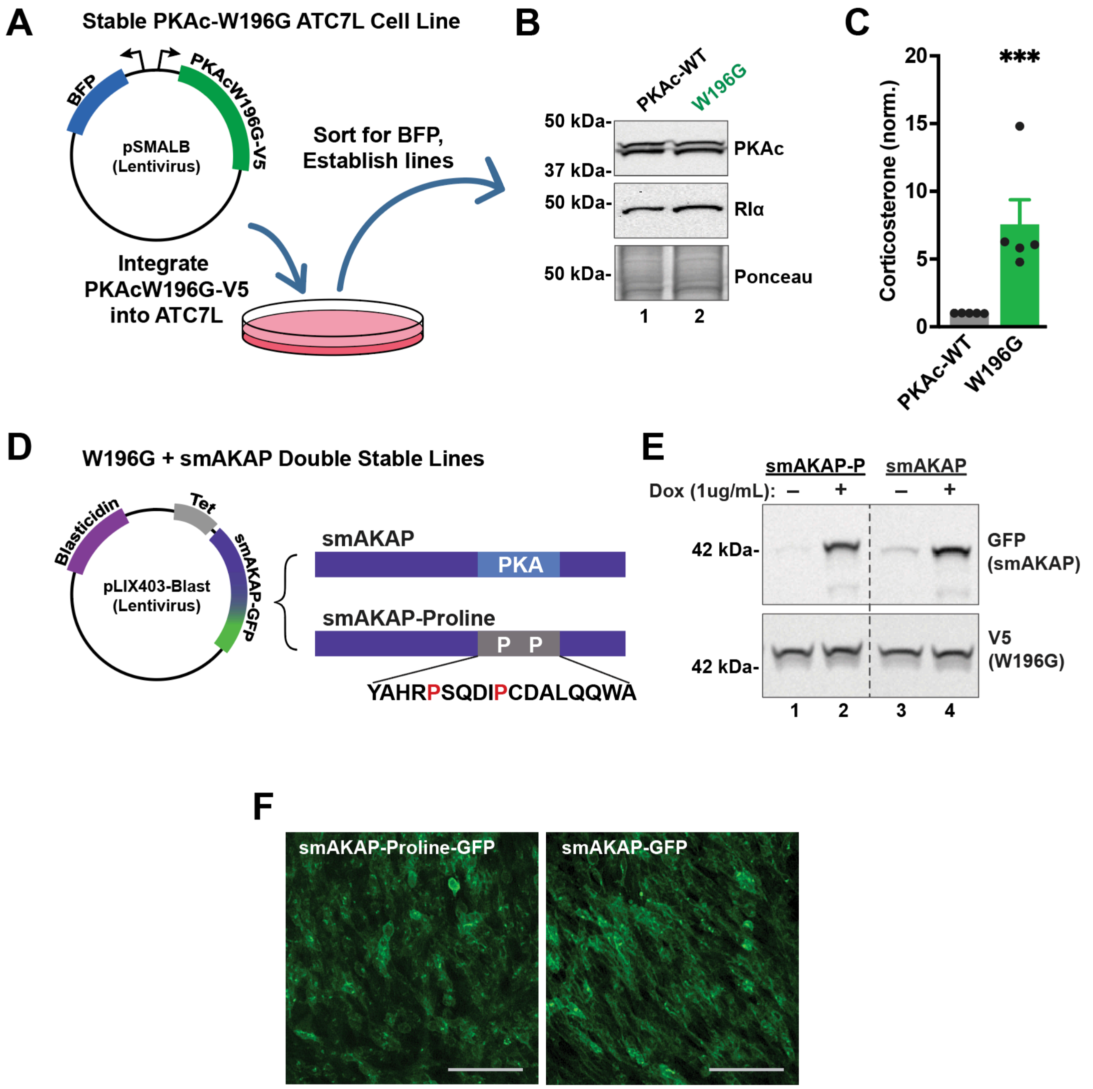

Figure S4: Generation and validation of stable adrenal cell lines

(A) Lentiviral WT PKAc-V5 or PKAc^{W196G}-V5 plasmids were integrated into ATC7L adrenal cells. FACS cell sorting resulted in population lines stably expressing each kinase variant.

(B) Immunoblot showing levels of PKA subunits expressed in stable lines. Doublet for PKAc shows V5-tagged viral construct (top band) and endogenous protein (lower band).

(C) Corticosterone meas. from stable ATC7L cell lines. Mean ± SE; ***p≤0.001, Student's t test.

(D) Lentiviral plasmid and linear protein constructs for smAKAP and smAKAP-proline control used to make double stable PKAc^{W196G}/smAKAP lines.

(E) Immunoblot showing PKAc^{W196G} expression and doxycycline-inducible expression of smAKAP constructs in double stable cell lines.

(F) Low magnification image of GFP signal from tagged smAKAP proteins in double stable ATC7L cell lines. Scale bar = 100 µm.

SUPPLEMENTAL MOVIE LEGENDS

Supplemental movie 1. H295R cells expressing AKAP79-YFP (cyan), RIIα-iRFP, and WT PKAc tagged with photoactivatable mCherry (magenta). Sampled at 2 Hz, with 2 seconds of baseline and 13 seconds of recording after photoactivation.

Supplemental movie 2. H295R cells expressing AKAP79-YFP (cyan), RIIα-iRFP, and PKAc-W196G tagged with photoactivatable mCherry (magenta). Sampled at 2 Hz, with 2 seconds of baseline and 13 seconds of recording after photoactivation.

Supplemental movie 3. H295R cells expressing smAKAP-GFP (yellow), RIα-iRFP, and WT PKAc tagged with photoactivatable mCherry (magenta). Sampled at 2 Hz, with 2 seconds of baseline and 13 seconds of recording after photoactivation.

Supplemental movie 4. H295R cells expressing smAKAP-GFP (yellow), RIα-iRFP, and PKAc-W196G tagged with photoactivatable mCherry (magenta). Sampled at 2 Hz, with 2 seconds of baseline and 13 seconds of recording after photoactivation.
